# Myeloid-Derived Suppressor Cells and Pancreatic Cancer: Implications in Novel Therapeutic Approaches

**DOI:** 10.3390/cancers11111627

**Published:** 2019-10-24

**Authors:** Anita Thyagarajan, Mamdouh Salman A. Alshehri, Kelly L.R. Miller, Catherine M. Sherwin, Jeffrey B. Travers, Ravi P. Sahu

**Affiliations:** 1 Department of Pharmacology and Toxicology, Boonshoft School of Medicine Wright State University, Dayton, OH 45435, USA; anita.thyagarajan@wright.edu (A.T.); alshehri.60@wright.edu (M.S.A.A.); jeffrey.travers@wright.edu (J.B.T.); 2 Department of Pharmacology and Toxicology, Pharmacy College, Taibah University, Medina 42353, Saudi Arabia; 3 Department of Internal Medicine, Boonshoft School of Medicine Wright State University, Dayton, OH 45435, USA; robbins.9@wright.edu; 4 Department of Pediatrics, Boonshoft School of Medicine Wright State University/Dayton Children’s Hospital, Dayton, OH 45404, USA; 5 Dayton Veteran’s Administration Medical Center, Dayton, OH 45435, USA

**Keywords:** myeloid-derived suppressor cells, pancreatic cancer, pancreatic cancer therapies

## Abstract

Pancreatic ductal adenocarcinoma (PDAC) remains a devastating human malignancy with poor prognosis and low survival rates. Several cellular mechanisms have been linked with pancreatic carcinogenesis and also implicated in inducing tumor resistance to known therapeutic regimens. Of various factors, immune evasion mechanisms play critical roles in tumor progression and impeding the efficacy of cancer therapies including PDAC. Among immunosuppressive cell types, myeloid-derived suppressor cells (MDSCs) have been extensively studied and demonstrated to not only support PDAC development but also hamper the anti-tumor immune responses elicited by therapeutic agents. Notably, recent efforts have been directed in devising novel approaches to target MDSCs to limit their effects. Multiple strategies including immune-based approaches have been explored either alone or in combination with therapeutic agents to target MDSCs in preclinical and clinical settings of PDAC. The current review highlights the roles and mechanisms of MDSCs as well as the implications of this immunomodulatory cell type as a potential target to improve the efficacy of therapeutic regimens for PDAC.

## 1. Introduction

Pancreatic ductal adenocarcinoma (PDAC) is an aggressive malignancy, which represents the fourth leading cause of cancer-related deaths in the United States [1,2,3]. A mere 2–9% of patients survive for five years with an average life expectancy of only six months for stage IV adenocarcinoma [3,4,5]. Several therapeutic agents have been explored with or without standard gemcitabine such as 5-fluorouracil (5FU), oxaliplatin, nab-paclitaxel, or FOLFIRINOX (a combination of 5FU, leucovorin, irinotecan, and oxaliplatin), which have resulted in marginal to a modest increase in the life span of PDAC patients [6,7,8,9,10,11,12,13]. While these therapeutic regimens remain commonly employed treatment options for pancreatic cancer, evidence from ongoing clinical studies indicates that the efficacy of such therapies could be hampered via mechanisms involving cellular resistance as well as the potential interference due to paradoxically promoting the immunosuppressive milieu of systemic and tumor microenvironment (TME) [14,15]. 

Importantly, several factors involved in the development of tumor resistance mechanisms, particularly those induced by TME-associated suppressive immunophenotypes such as myeloid-derived suppressor cells (MDSCs) and regulatory T cells (Tregs) have been extensively studied [15,16,17] as they play critical roles in impacting anti-tumoral immune responses [18,19,20,21]. Therefore, substantial efforts have been directed towards exploring the mechanisms by which such immunophenotypes occur, to design potentially targetable approaches for the treatment of malignancies including PDAC [22,23,24,25,26]. 

MDSCs are a heterogeneous population of immature myeloid cells, implicated in most of the pathological conditions, including obesity, autoimmunity, chronic inflammation, trauma, and cancer progression [27,28,29]. Growing evidence indicates that MDSCs accumulate and expand in the peripheral blood or other organs (e.g., spleen, liver, and lung) and at tumor sites, where they modulate host antitumor immune responses [18,19,21,30]. In general, MDSCs-induced immunosuppression is mediated via the orchestration of multiple signaling pathways, interactions with several immune cells and mediators, which directly or indirectly not only suppress anti-tumoral immunity and favor cancer progression, angiogenesis, and metastasis but also impede the efficacy of therapeutic agents [18,19,20,21,30]. Thus, MDSCs can pose serious challenges in the treatment of PDAC.

Different subsets of MDSCs have been evaluated to correlate their levels or frequencies in peripheral blood and tumor tissue with PDAC development. While MDSCs levels do not always reveal a definitive association with tumor stages in pancreatic cancer patients [15,30], some studies have shown a positive correlation between MDSCs and PDAC progression. Notably, increased pro-MDSCs cytokines were detected at higher levels in chemotherapy-treated compared to chemo-naive patients and healthy donors [30]. Overall, such findings not only validate the role of MDSCs in pancreatic cancer but also indicate that its levels may be used as predictive biomarkers of chemotherapy failure. Of significance, therapeutic agents targeting MDSCs have been shown to restore anti-tumoral immunity and/or enhance the efficacy of immunotherapy against malignancies, including PDAC [14,22,24]. The roles and mechanisms of MDSCs, as well as its implications as a potential target for therapeutic regimens against pancreatic cancer, are discussed below.

## 2. Differentiation, Characteristics, and Mechanisms of MDSCs Function

**Figure 1:**
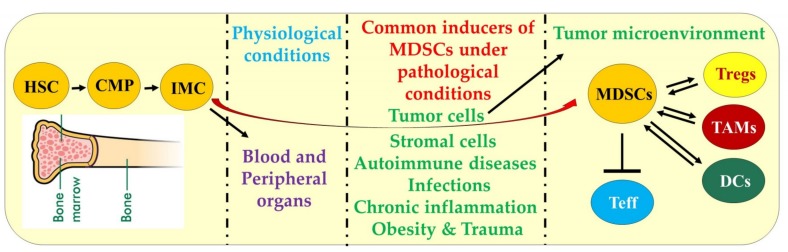
Differentiation of MDSCs. The generation of immature myeloid cells (myelopoiesis) begins in the bone marrow. The hematopoietic stem cells (HSC) differentiate into common myeloid progenitor (CMP) cells and then to immature myeloid cells (IMC). IMCs migrate to blood and various peripheral organs and under normal physiological conditions, differentiate into granulocyte- macrophage progenitors (GMP) cells to further differentiate into other progenitor cells and lineage-specific cell populations. Under pathological conditions including cancer, MDSCs are generated and acquire immunosuppressive functions. These MDSCs inhibit the antitumor immunity of effector T cells (Teff) directly or via mechanisms including the crosstalk with other immunosuppressive cell types such as regulatory T cells (Tregs), tumor-associated macrophages (TAMs) and dendritic cells (DCs).

The generation of immature myeloid cells (IMC) occurs in the bone marrow as a part of the normal process of myelopoiesis, and is regulated by a complex network of soluble factors including granulocytes macrophage colony-stimulating factor (GM-CSF), granulocyte colony-stimulating factor (G-CSF) and macrophage colony-stimulating factor (M-CSF) [31,32,33,34]. In this process, the hematopoietic stem cells (HSC) differentiate into common myeloid progenitor (CMP) cells and then to immature myeloid cells (IMCs) (Figure 1). These IMCs migrate to blood and various peripheral organs and under normal physiological conditions, differentiate into granulocyte/macrophage progenitors (GMP) cells. These GMPs further differentiate into monocytic/dendritic progenitor (MDP) cells or myeloblasts (MB) to further differentiate and develop into lineage-specific cell populations such as granulocytes, macrophages, or dendritic cells (DCs), respectively. However, in pathological conditions including cancer, the overproduction of these soluble factors favor MDSCs generation. In cancer models, MDSCs within the tumor microenvironment can crosstalk with other immune cell types such as Tregs, TAMs and DCs and inhibit effector T cells (Figure 1). MDSCs, based on their phenotypic and morphological features or cell surface markers (Table 1), can be characterized into two major types: Polymorphonuclear (PMN-MDSCs) or granulocytic (G-MDSCs or Gr-MDSCs) and mononuclear or monocytic (M-MDSCs or Mo-MDSCs) [29,35,36,37]. While murine G-MDSCs can be identified via CD11b^+^Gr-1^+^Ly6G^high^Ly6C^low^ and M-MDSCs by CD11b^+^Gr-1^+^Ly6G^low/–^Ly6C^high^ markers, human G-MDSCs express HLA-DR-CD33^+^CD11b^+^CD15^+^CD14^−^ and M-MDSCs express HLA-DR^low^-CD11b^+^CD14^+^CD15^−^ markers. However, these MDSCs subsets in humans can also be complemented by various other markers, but lack Gr-1 expression [24,35,36,37]. Notably, high and intermediate levels of Gr-1 (Gr-1^high^ or Gr-1^int^) and Ly6C (Ly6C^high^ or Ly6C^int^) have also been indicated to potentially distinguish between G-MDSCs and M-MDSCs subsets in TME [24]. Also, G-MDSCs can be better represented as PMN-MDSCs as these cell types are distinct from the steady-state neutrophils in several phenotypic characteristics including having fewer granules, reduced CD16 and CD62L expression, and increased levels of arginase 1 and peroxynitrite [24,35,36,37]. Of note, the phenotype for early-stage MDSCs (eMDSCs) in human peripheral blood mononuclear cells (PBMCs) is defined by Lin^−^(CD3/14/15/19/56)/HLA-DR^−^/CD33^+^, but eMDSCs markers for murine cells have not been determined [34,35,36,37]. 

**Figure 2:**
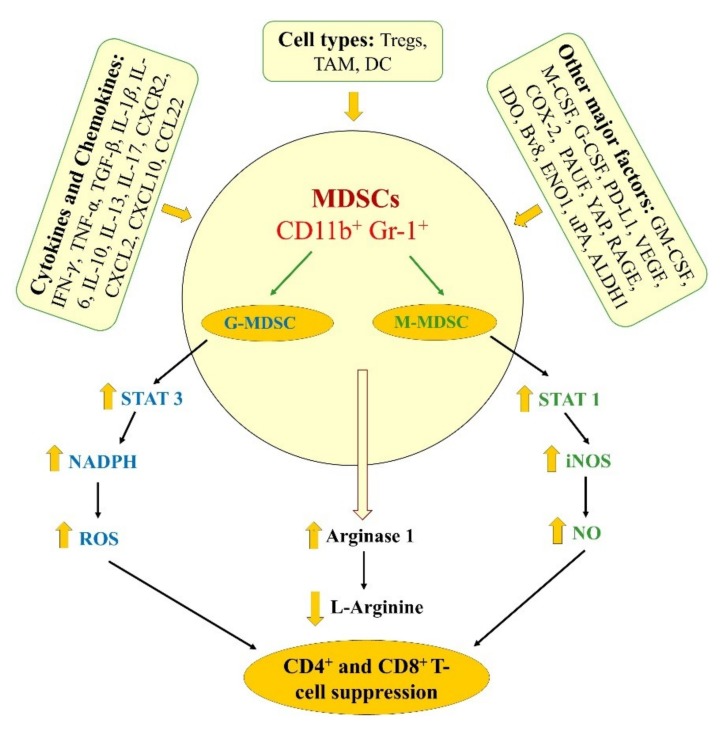
Mechanisms involve in mediating the immunosuppressive function of MDSC, which include various cytokines and chemokines, immune cells and major factors, implicated in inhibiting T-cell responses as it related to tumorigenesis.

Thus far, several functional assays have been explored to precisely define the ability of MDSCs, primarily implicated in the suppression of various immune cell types with the gold standard being the evaluation of the inhibition of T-cell proliferation or activity [35,36,37]. Given that MDSCs not only exhibit differences in their phenotypic and morphological characteristics but also acquire distinct functional heterogeneity, it is essential to validate their mechanisms to precisely characterize their roles in disease related pathophysiologies including tumor progression. Figure 2 depicts an underlying mechanisms (i.e., cell types, cytokines, chemokines, growth and angiogenic factors, and immune mediators) involved in modulating MDSCs function and expansion directly or indirectly [18,19,21,24,36,37], and the related studies in pancreatic cancer models are discussed below.

Notably, in most tumor models, G-MDSCs predominate (70–80% or higher) with lesser amounts of M-MDSCs (20–30%) of total MDSCs population. G-MDSCs usually require activation of the signal transducer and activator of transcription 3 (STAT3) and increased NADPH oxidase activity, which results in increased reactive oxygen species (ROS) generation, but less nitric oxide (NO) production [29,35,36]. These cellular events lead to the post-translational modification of T-cell receptors and T-cell unresponsiveness, which contributes to the suppression of adaptive anti-tumoral immunity (i.e., CD4^+^ and CD8^+^ T cells; Figure 2) [29,35,36]. On the other hand, M-MDSCs often have increased expression of STAT1 and inducible nitric oxide synthase (iNOS) as well as higher levels of NO (produced via iNOS-mediated L-arginine metabolism). These changes result in the suppression of T-cell responses both in antigen-specific and nonspecific manner via mechanisms including the inhibition of Janus kinase 3 (JAK3), STAT5, and MHC class II expression, and induce T-cell apoptosis [27,28,29]. However, activation of STAT3 signaling has also been shown to mediate M-MDSCs function, which also exhibit less ROS production compared to G-MDSCs [27,28,29]. Importantly, both MDSC subsets have been shown to exhibit increased levels of arginase 1 activity that induces T-cell suppression via the depletion of L-arginine [27,28,29]. While several studies supported the requirement or induction of arginase 1 expression in MDSCs function, a recent study opposed this fact via experimental evidences indicating that arginase 1 is neither constitutively expressed in nor mediate the immunosuppressive or inhibition of T-cell proliferation effects of MDSCs. [38]. Overall, the data demonstrated that arginase 1 expression in MDSCs can be induced via approaches including exposure to TCR-activated T cells, which is dependent upon the two signaling-relay axes, IL-6-to-IL-4 and GM-CSF-IL-4-to-IL-10 but is independent of arginase 1 activity [38].

## 3. Preclinical Studies Delineating the Significance of MDSCs in Pancreatic Cancer and Therapies

**Figure 3:**
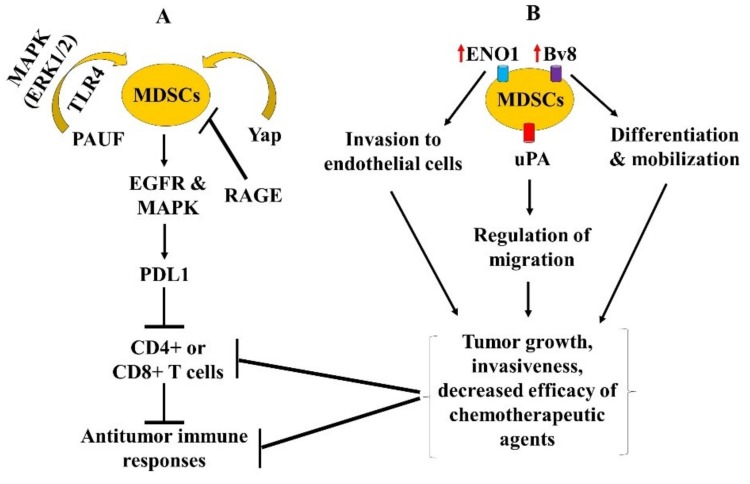
Schematic representations of A) other signaling pathways and B) factors involved in the regulation of MDSCs differentiation, accumulation, migration and function. The mediators (i.e., cytokines, chemokines, angiogenesis and immune cells) involved in the context of these pathways or factors, have been summarized in the text and also included in Figure 2, thus, are not repeated here. The sign → denotes induction or regulation, 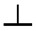 inhibition and 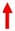 increase or upregulation.

Several studies have highlighted the roles of MDSCs in masking the anti-tumor immune responses using in vivo pancreatic cancer models (summary in Table 2 and Figure 3A,B).

### 3.1. Genetically Engineered Mouse Models of Pancreatic Cancer in MDSCs Studies

A recent report defined the roles of myeloid cell subsets on the onset and progression of a pancreatic tumor in the context of T-cell-mediated immunity using a syngeneic transplantation model and genetically engineered mouse models (GEMM) where CD11b-DTR mice were backcrossed with mice harboring genetic alterations in Kras/p53/p48 genes [19]. This system facilitated the depletion of all CD11b^+^ myeloid cell subsets including MDSCs upon the treatment with diphtheria toxin (DT), which prevented the formation of pancreatic intraepithelial neoplasia (PanIN) during the initiation phase of pancreatic carcinogenesis, and also caused tumor growth arrest or tumor regression of pre-established tumors [19]. These effects were mediated via increased tumor cell apoptosis, which was dependent on tumor-infiltrating CD8^+^ T cells, indicating that myeloid cells serve to negatively regulate T-cell dependent anti-tumor immunity. Also, reduced expression of immune checkpoint ligand PD-L1 was noted in CD11b^+^ myeloid cells depleted mice, and its expression was found to be regulated via mechanisms involving the activation of epidermal growth factor receptor (EGFR) and mitogen-activated protein kinases (MAPK) pathways [19]. Altogether, these findings indicated that myeloid cells favor tumor immunoevasion in a process dependent on EGFR and MAPK-mediated regulation of tumoral PD-L1 expression and inhibition of CD8^+^ T cell antitumor immunity. 

A study led by Stromness and colleagues determined that targeting MDSCs in an autochthonous GEMM exhibits the potential to unmask or restore the ability of adaptive immune responses to target PDAC [24]. Their findings demonstrated that CD4^+^FoxP3^+^ Tregs and tumor-associated macrophages (TAM) were predominantly found at the initial (preinvasive) stage of PDA. However, the infiltration of CD11b^+^Gr-1^+^ MDSCs (abundantly G-MDSCs) was markedly increased during the transition from preinvasive to invasive stages of PDA development, and that GM-CSF was found to be crucial in enhancing the survival of G-MDSCs, which inhibited T-cell proliferation and induced T-cell apoptosis [24]. G-MDSCs depletion unmasked PDAC to adaptive immune responses that resulted in increased activation and infiltration of CD8^+^ T cells into the stroma, and tumor epithelial cells, inducing increased apoptosis of tumor epithelial cells [24]. Some of these findings were supported by other studies in elastase-transforming growth factor-alpha (EL-TGF-α)/p53^−/−^ spontaneous pancreatic tumor model [39]. Zhao and colleagues demonstrated an increased frequency of MDSCs in the blood, lymphatic organs and pancreas during early stages of tumor development, which further increased upon tumor progression in EL-TGF-α/p53^−/−^ mice compared to EL-TGF-α/p53^+/−^ mice with premalignant lesions, and wild-type (WT) mice harboring subcutaneous mPAC tumors [39]. These MDSCs were immunosuppressive in nature as measured by increased arginase 1 activity as well as suppression of T-cell proliferation and inhibition of IFN-γ secretion in EL-TGF-α/p53^−/−^ mice compared to EL-TGF-α/p53^+/−^ and WT mice. Overall, these findings highlighted the significance of myeloid cells at the early stages of pancreatic tumor development, and its blockade to achieve sustained T-cell dependent antitumor immune responses.

Moreover, the receptor for advanced glycation end products (RAGE), a pattern recognition receptor associated with damage-associated molecular pattern molecules, is overexpressed in both the human and murine PDA models [40,49,50]. To define the role of RAGE, Vernon and colleagues utilized a spontaneous murine triple transgenic model of PDA in a RAGE-null background (i.e., KCR mice) and demonstrated that RAGE ablation was associated with delayed pancreatic carcinogenesis [40]. Decreased MDSCs frequency was noted in KCR mice compared to the mice exhibiting mutant Ras-promoted pancreatic carcinogenesis (i.e., KC mice), RAGE-null (RAGE^−/−^), and wild type mice [40]. Importantly, KRC mice exhibited a mature, but non-immunosuppressive splenic myeloid (CD11b^+^Gr-1^−^F4/80^+^) phenotypes, which were characterized by decreased expression of CCL22 to CXCL10 in pancreatic tissues, as well as reduced serum IL-6 levels compared to the KC mice [40]. These findings indicated that RAGE plays an essential role in the regulation of immunoregulatory milieu within TME during pancreatic carcinogenesis. Similarly, yes-associated protein (Yap) has also been identified to be a critical regulator of immunosuppressive TME in both mouse and human PDAC. Yap induces the expression, and secretion of cytokines and chemokines involved in the differentiation and accumulation of MDSCs in both the splenic and TME (Figure 2), in the process blocked by its ablation [51]. 

Cancer stem cells (CSCs) are the subpopulation of tumor cells, which have the capability of inducing tumor initiation as well as tumor resistance to therapeutic agents [52,53,54]. Thus far, among several markers used to characterize CSCs, aldehyde dehydrogenase-1 (ALDH1), an intracellular detoxifying enzyme has been extensively used to identify CSCs in pancreatic cancer. PDAC patients with increased ALDH1^+ or Bright^ expressing CSCs have been shown to be associated with decreased progression-free and overall survival [55,56]. While multiple cell types within the TME have been demonstrated to promote the stemness of CSCs in murine pancreatic cancer models, studies by Panni and colleagues have provided important insights as to how the levels of ALDH1 are regulated [41]. As G-CSF (i.e., CSF-3) is critical for myeloid cell production, the authors utilized a G-CSF receptor knockout (GCSFR^−/−^) mouse model with transplantable murine pancreatic cancer cell lines, derived from the spontaneous pancreatic cancer arising from GEMM or chemical carcinogenesis-induced mice models [41]. While MDSCs from the tumor-bearing GCSFR^−/−^ mice exhibited similar immunosuppressive properties as found in tumor-bearing WT mice, GCSFR^−/−^ mice had reduced bone marrow myelopoiesis, and decreased levels of G-MDSCs and M-MDSCs in peripheral blood and tumors [41]. These effects in GCSFR^−/−^ mice were associated due to a shift in TH-2 to TH-1 immune responses as characterized by increased expression of IFN-γ, TNF-α, and IL-12 and decreased expression of ARG-1, TGF-β, IL-6, and IL-10 as well as reduced ALDH1^Bright^ CSCs. Enhanced STAT3 signaling in M-MDSCs was found to promote CSCs and epithelial to mesenchymal transition (EMT) in this murine pancreatic cancer model [41].

### 3.2. Immune Mediators Affecting MDSCs Function, and Approaches Targeting MDSCs in Mice Models Including GEMM

Several immune mediators have been demonstrated to affect the accumulation of tumoral and systemic MDSCs and its immunosuppressive function. A study by Karakhanova and colleagues reported significantly increased levels of tumoral vascular endothelial growth factor (VEGF) as well as tumoral and lymphatic MDSCs in the murine panc02 orthotopic pancreatic cancer model as compared to pancreas tissues, which was correlated with increased tumor growth [42]. Only MDSCs, but not dendritic cells and macrophages were found to exhibit immunosuppressive functions as measured by increased expression of arginase-1 and iNOS in MDSCs as well as the decreased percentage of CD4^+^ and CD8^+^ T cell proliferation with the addition of MDSCs. Moreover, increased levels of inflammatory cytokines and factors including TNF-α, IL-6, IL-13, VEGF, TGF-β, IL-1β, and IL-17 were detected in the co-culture supernatants of splenocytes and MDSCs, indicated that chronic inflammatory milieu favor MDSCs expansion and accumulation [42]. Systemic treatment of sildenafil, a phosphodiesterase-5 inhibitor, resulted in decreased levels of VEGF, MDSCs, and Tregs as well as increased tumor infiltration of CD4^+^ T cells, but not CD8^+^ T cells, which also increased the survival of only tumor-bearing female mice [42]. While a gender-specific discrepancy in sildenafil efficacy in prolonging the survival of female mice over male mice was not mechanistically apparent, these findings revealed the importance of chronic inflammation and angiogenic factor in influencing MDSCs frequency, and thus, favoring tumor growth. 

Another study determined the significance of pancreatic adenocarcinoma up-regulated factor (PAUF) and demonstrated that PAUF-overexpressing pancreatic cancer cells promoted the accumulation of MDSCs in both the splenic and pancreatic TME. The targeted depletion of PAUF via shRNA and antibody-mediated approaches inhibited, and PAUF overexpression enhanced the accumulation and immunosuppressive activity of MDSCs [43]. These effects were mediated via toll-like receptor 4 (TLR-4) signaling in a process blocked by neutralizing antibodies against TLR4 [43]. Also, increased expression of gene-encoding factors involved in the activation of MDSCs including ARG-1, cyclooxygenase type 2 (COX-2), iNOS2 and cytochrome b-245, beta polypeptide (Cybb) were detected by PAUF overexpression. Importantly, PAUF-induced increased expression of these genes were found to be dependent on AP-1 transcription factors in the process significantly blocked by MAPK, particularly ERK inhibition [43]. Overall, these data indicated the PAUF is a tumor growth promoter that regulates MDSCs. Thus, targeting PAUF has therapeutic potential in pancreatic cancer.

Targeting MDSCs has been explored as one of the potential therapeutic approaches in pancreatic cancer models (summary in Table 2). The effects of several chemotherapeutic agents, namely cyclophosphamide, doxorubicin, oxaliplatin, paclitaxel, gemcitabine, 5FU, and raltitrexed have been studied in antitumor immune responses and the relevance of MDSCs in this context [44]. Using the murine EL4 thymoma model, Vincent and colleagues demonstrated increased frequency of MDSCs in the splenic population and tumor beds before the treatments with chemotherapeutic agents, yet only gemcitabine and 5FU were found to significantly reduced MDSCs frequency in splenic and pancreatic TME [44]. Notably, 5FU exhibited increased cytotoxic effects towards MDSCs as compared to gemcitabine that resulted in an enhanced infiltration of tumor-specific CD8^+^ T cells as well as increased production of IFN-γ by CD8^+^ T cells. These changes promoted the anti-tumor immune response that resulted in tumor growth suppression in syngeneic C57BL/6 (WT) mice. However, only a minor effect was noted in tumor growth suppression in T-cell deficient nude mice, indicating that 5FU predominantly exerted its effects via its ability to deplete MDSCs [44]. Importantly, a regimen of 5FU combined with cyclophosphamide that depletes the Tregs population exerted a synergistic T-cell dependent anti-tumor response in WT mice compared to nude mice [44]. Similarly, Hasnis and colleagues demonstrated that weekly gemcitabine treatment combined with metronomic chemotherapy (MC, i.e., daily administration in low doses) with gemcitabine resulted in delayed pancreatic tumor regrowth, inhibition of tumor metastasis, and tumor infiltration of MDSCs compared to weekly gemcitabine alone in an orthotopic pancreatic cancer model [45]. Notably, enhanced tumor angiogenesis and tumor mobilization of MDSCs were observed with weekly gemcitabine alone treatment, which was associated with increased expression of prokineticin 2 (PK2 or Bv8, a factor involved in MDSCs differentiation and mobilization) compared to MC gemcitabine or the combination of these regimens [45]. Blockade of Bv8 enhanced the efficacy of weekly gemcitabine treatment in terms of reversing these effects and increasing mice survival, indicating that approaches targeting Bv8 could be exploited to prevent chemotherapy-induced MDSCs mobilization in pancreatic cancer. 

An increased prevalence of PDA-associated antigen α-enolase (ENO1) has been documented in pancreatic cancer patients (i.e., in 60% cases), which correlated with a better prognosis [57]. ENO1 DNA vaccination has been shown to elicit robust anti-tumor immune response as well as increased survival of pancreatic cancer-bearing GEMM mice [58]. A recent study determined the effects of ENO1 on MDSCs functions and effector T-cell responses in a syngeneic transplantable pancreatic cancer model [46]. Cappello and colleagues observed increased frequency of ENO1-expressing MDSCs in the blood of PDA patients compared to healthy donors as well as tumor-bearing KC mice compared to the age-matched littermates (control mice) [46]. Monoclonal antibody targeting ENO1 impaired MDSCs invasion to endothelial cells without significantly affecting its immunosuppressive function. This anti-ENO1 treatment also decreased Treg expansion, associated with increased IL-6 and decreased TNF-α secretion as well as decreased ARG-1 activity. Together indicating that anti-ENO1 impacts Th17 differentiation and elicits sustained T-cell effector function [46]. Importantly, increased IFN-γ and IL-17 as well as decreased IL-10 and TGF-β secretion was observed by activated T cells in the presence of anti-ENO1 treated MDSCs compared to the control MDSCs. These findings indicated the potential of targeting ENO1 in immunotherapy approaches against pancreatic cancer. 

Similarly, CXCR2 signaling, a G-protein-coupled receptor for human CXC chemokines including CXCL1, CXCL2, CXCL3, CXCL5, and CXCL8, implicated in the regulation of neutrophils and migration of MDSCs, appears to play a critical role in the invasiveness, metastatic potential, or poorer prognosis in the preclinical and clinical settings of malignancies including pancreatic cancer [47,59,60,61,62,63,64]. Inhibition of CXCR2 signaling has been shown to disrupt tumor-stromal interactions and improves survival in murine PDA models [63]. Importantly, studies by Steele and colleagues have demonstrated that CXCR2 deletion abrogated tumor metastasis in KPC Cxcr2^−/−^ mice, yet, no benefits were noted in the overall or tumor-free survival between KPC Cxcr2^−/−^ and KPC mice with or without gemcitabine treatment [47]. As neutrophils and MDSCs are the most prominent sources of CXCR2 in mice, the deletion of Ly6G^+^ cells in KPC mice resulted in reduced metastasis. No effects were noted in the host survival, indicating that Ly6G^+^ deletion mimics the effects of Cxcr2 deletion. In contrast, treatment with a clinically relevant small molecule inhibitor of CXCR2 in KPC mice not only reduced tumor metastasis but also prolonged mice survival with the combination of CXCR2 inhibitor and gemcitabine or anti-PD-1 immunotherapy [47]. Importantly, increased tumor infiltration of both CD4^+^ and CD8^+^ T cells were noted in mice treated with CXCR2 inhibitor and anti-PD-1, indicating that targeting CXCR2 might have the therapeutic potential in the pre-metastatic setting and could also enhance the efficacy of chemotherapy and immunotherapy against pancreatic cancer [47]. 

Similarly, increased serine proteases, particularly, urokinase-type plasminogen (uPA) and its receptor have been correlated with increased migration of inflammatory cells, pancreatic cancer growth, and invasiveness as well as poor outcomes [65]. The effects of myxomaviral anti-inflammatory proteins, Serp-1, which inhibits uPA, plasmin and coagulation factor X, and M-T7 that inhibits C, CC, and CXC chemokines are implicated in the regulation of MDSCs in cancer models including pancreatic cancer [48]. Treatments with Serp-1 and neuroserpin (a mammalian serpin and inhibitor of thrombolytic proteases), but not M-T7 were found to specifically reduce the in vivo proliferation and growth of pancreatic tumor xenografts. These effects were mediated via a significant decrease in both the splenic and tumoral MDSCs frequency and reduced tumor infiltration of macrophages, indicating its potential therapeutic efficacy in pancreatic cancer treatment [48].

## 4. Clinical Studies Delineating the Role of MDSCs in Pancreatic Cancer and Therapies

Multiple clinical studies have evaluated MDSCs in PDAC patients and also explored approaches targeting MDSCs with or without therapeutic agents in this population. These studies are discussed below and also summarized in Table 3.

### 4.1. Characterization of MDSCs in PDAC Patients

Khaled and colleagues characterized MDSCs subsets to evaluate their levels and functions in the blood and tumor tissues of pancreatic cancer patients [15]. They demonstrated that the most dominant phenotype in the circulation (i.e., PBMCs) and tumor-infiltrating pancreatic tissues were G-MDSCs (Lin-HLA-DR-CD33^+^CD11b^+^CD15^+^) and not M-MDSCs (Lin-HLA-DR-CD14^+^) when compared with chronic pancreatitis patients and healthy donors [15]. The functional activity of these circulating G-MDSCs was assessed by arginase 1 expression. Nevertheless, this increased G-MDSCs was not correlated with pancreatic cancer stages, indicating that they play essential roles in pancreatic cancer, yet warranted a need for further validation studies in a large cohort of patients to guide the development of new approaches to target MDSCs. In contrast, in a separate report, increased frequency of MDSCs (CD11b^+^CD15^+^) in the peripheral circulation and bone marrow was correlated with increasing disease stages in pancreatic cancer patients compared to normal controls [66]. Also, compared to the normal healthy pancreas, increased numbers of tumor-infiltrating MDSCs co-expressing CD15 and arginase-1 were observed in patients, validating the importance of MDSCs in pancreatic cancer progression [66].

In another report, Trovato and colleagues analyzed the frequencies of monocytes (CD14^+^CD15^−^CD11b^+^), granulocytes (PMNs, CD15^+^CD14^−^CD11b^+^), MDSC1 (CD14^+^IL-4Rα^+^), MDSC2 (CD15^+^IL-4Rα^+^), MDSC3 (Lin^−^HLA-DR^−^CD33^+^), and MDSC4 (CD14^+^HLA-DR^−/low^) subsets in the fresh whole blood and frozen PBMCs of PDAC patients of three independent cohorts [67]. The authors observed significantly increased frequency of CD14^+^ cell phenotype representing heterogeneous population of M-MDSCs. Importantly, M-MDSCs exhibited distinct cytological features (smaller in size), immune suppressive properties and gene signatures with activated STAT3 and ARG1 pathway. These M-MDSCs were associated with metastatic disease and a shorter overall survival of PDAC patients [67].

### 4.2. Effects of Therapeutic Agents on MDSCs Levels in PDAC Patients

In addition to chemotherapeutic agents used for treating solid tumors, administration of cytokines such as IL-2 and IFN-α or adoptive immunotherapy, including cytokine-induced killer cells (CIK) have been associated with better outcomes [72,73,74,75]. While CIK immunotherapy has been used as promising adoptive immunotherapy over combined IL-2 and IFN-α treatment, its efficacy has been hampered due to the interference of several factors including MDSCs [76]. To that end, Wang and colleagues evaluated the effects of CIK immunotherapy in combination with chemotherapeutic agents such as gemcitabine and 5FU that reduce MDSCs, to determine whether these combinations would enhance CIK efficacy against solid tumors, including pancreatic cancer [14]. The pancreatic cancer patients were treated with CIK and CIK plus gemcitabine, and 5-FU and peripheral blood (pre- and post-treatment) was analyzed for MDSCs levels, and their response was assessed for overall survival. Both these chemotherapies decreased the frequency (%) of MDSCs (HLA-DR^+^CD11b^+^CD33^+^) compared to the pre-treatment and that the combination of CIK and chemotherapy was associated with increased survival of metastatic renal cell carcinoma and pancreatic cancer patients compared to CIK alone treated patients [14]. These findings suggest that MDSCs-targeting chemotherapy improves the survival response of CIK immunotherapy. 

Another study evaluated the levels of pro-MDSC cytokines including (PDGF-bb, FGF2, VEGFA, IL-4, IL-6, IL-8, IL-17, CCL5, and S100A9) and MDSCs using a five antigen panels (CD33, HLA-DR, CD11b, CD14, and CD15) in PDAC patients undergoing chemotherapy [30]. Markowitz and colleagues demonstrated increased levels of MDSCs in PBMCs of patients with progressive disease compared to patients with stable disease [30]. Also, increased levels of these pro-MDSC cytokines in the plasma were observed in chemo-naive patients compared to the healthy donors. Importantly, patients with stage III or IV PDAC undergoing chemotherapy exhibited increased levels of IL-6, which correlated with disease progression. These findings suggest that the analysis of MDSCs in peripheral blood may represent a predictive biomarker for chemotherapy failure in pancreatic cancer patients. A recent study evaluated the safety and feasibility of preoperative zoledronic acid (ZA) as neoadjuvant therapy in respectable PDAC patients [68]. Also, the patients’ blood and bone marrow were tested before and three months after the surgery to evaluate the prevalence of G-MDSCs and to correlate with the overall survival (OS) and progression-free survival (PFS) [68]. The authors demonstrated that the median OS of 18 months and PFS of 12 months were not correlated with G-MDSCs levels in pre-and post-ZA treated patients. However, common grade 1 or 2 toxicities including anorexia and arthralgia were noted with ZA treatment. These studies indicated that ZA treatment as a neo-adjuvant therapy did not have any significant impact on G-MDSCs in PDAC patients, yet the G-MDSCs frequency was found to be decreased in the blood in murine pancreatic cancer studies [68].

In another report, the effects of combining gemcitabine and capecitabine along with the class II telomerase peptide vaccine (GV1001) were evaluated on the dynamics of MDSCs (Lin-DR-CD11b^+^) in advanced-stage pancreatic cancer patients [69]. In this clinical setting, 19 patients received gemcitabine and capecitabine alone (arm two) and 21 patients received GV1001 vaccination concurrently with gemcitabine and capecitabine with a low-dose GM-CSF as an adjuvant (arm three). Briefly, eight out of 19 (42%) patients in arm two exhibited significantly decreased levels of MDSCs. However, among seven out of 19 patients with progressive disease (PD), five patients had increased and two patients had reduced levels of MDSCs. Among 10 patients with stable disease (SD), six patients had increased and four patients had reduced levels of MDSCs [69]. MDSCs levels pre-treatment were higher for arm three compared to arm two. Importantly, in arm three, nine out of 21 patients who developed an immune response to GV1001 administered concomitantly with gemcitabine and capecitabine, eight patients had decreased MDSCs levels. Overall, a significant decrease in MDSCs levels (18 out of 21 patients) post-treatment was noted in arm three patients compared to pre-treatment, whereas no significant changes were observed between pre-treatment and post-treatment in arm two patients [69]. There was no significant change in MDSCs levels between arm two and arm three post-treatment. These findings indicate that gemcitabine and capecitabine did not result in consistent reduction in MDSCs level and that high levels of MDSCs pre-vaccination do not necessarily prevent development of an immune response to tumor antigens.

As an inflammatory milieu can affect MDSCs function, anti-inflammatory triterpenoid has been shown to block the immunosuppressive function of MDSCs and/or improve immune response in both the human and mouse models of cancers including pancreatic cancer [70,77]. Nagaraj and colleagues reported the effects of synthetic triterpenoid derivative CDDO-Me on MDSCs frequency and immune response with or without gemcitabine in human and mouse models of pancreatic cancer [70]. In this clinical study, locally advanced and metastatic PDAC patients were treated with CDDO-Me (RTA-402) alone or in combination with gemcitabine, and PBMC (pre- and post-treatment) were assessed for MDSCs (Lin^−^HLA-DR^−^CD33+ and CD14^−^CD11b^+^CD33^+^). The data demonstrated that CDDO-Me completely abrogated the inhibitory effect of MDSCs, and no toxicity to CDDO-Me was observed. Also, while CDDO-Me and gemcitabine combination did not significantly affect the proportion of MDSCs, a significant increase in the patients’ T-cell responses to tetanus toxoid and PHA was noted [70]. These findings indicated that CDDO-Me has the potential to be used as a promising therapeutic option for immunotherapy due to its ability to block the immunosuppressive effects of MDSCs and improve immune response.

MDSCs can also be targeted via death receptors such as TNF-related apoptosis-induced ligand receptors (TRAIL-R), members of the TNF receptor superfamily, implicated in apoptotic pathways and play crucial roles in cancer initiation [78,79,80]. Importantly, TRAIL-R-2 agonistic antibody has been used as a targeted approach to selectively deplete MDSCs in a murine cancer model [80]. Using a similar approach, the same group tested the hypothesis if TRAIL-R2 antibody (DS-827a) can eliminate MDSCs in patients with advanced-stage solid tumors, including pancreatic cancer [71]. The peripheral blood and tumor tissues were evaluated for myeloid and lymphoid cell populations, which revealed that most of the DS-827a-treated patients had reduced numbers of MDSCs in peripheral blood compared to the samples obtained from pre-treatment and healthy donors [71]. However, in tumor samples decreased MDSCs was noted in 50% of the patients [71]. Other than MDSCs subsets, DS-827a did not affect neutrophil and monocyte counts as well as other populations of myeloid and lymphoid cells, suggesting its specificity towards MDSCs and its potential use in combination immunotherapy for cancer treatment. 

## 5. Conclusions

Preclinical and clinical studies suggested that several cytokines/chemokines, immune cells, signaling pathways, and other factors play crucial roles in regulating the immunosuppressive function of MDSCs. MDSCs are one of the major factors responsible for impacting pancreatic cancer progression, invasiveness, metastasis, and survival. MDSCs also impede antitumor immune responses of therapeutic agents in human and mouse models of pancreatic cancer. Thus, MDSCs could be used as a potential biomarker for not only assessing the tumor progression or tumor stages but also to define it they are the critical factor in the failure of chemotherapy or immunotherapy approaches in pancreatic cancer patients. To that end, targeting MDSCs has emerged as a promising approach in enhancing the efficacy of standard therapeutic agents, including immunotherapy. Moreover, as new interventions are still ongoing to precisely understand the exact mechanism of MDSCs function that governs its immunosuppressive activity, the implications of such new mechanisms in preclinical and clinical studies would provide a breakthrough in the treatment of cancer.

## Figures and Tables

**Table 1:** Summary of commonly expressed markers in human and murine MDSCs.

MDSCs	Common PMN-MDSCs Markers	Common M-MDSCs Markers	eMDSCs
Human	HLA-DR-CD33^+^CD11b^+^CD15^+^CD14^−^	HLA-DR^low^-CD11b^+^ CD14^+^ CD15^−^	Lin^−^(CD3/14/15/19/56)/HLA-DR^−^/CD33^+^
Murine	CD11b^+^Gr-1^+^Ly6G^high^Ly6C^low^	CD11b^+^Gr-1^+^Ly6G^low^Ly6C^high^	—

**Table 2:** Summary of preclinical studies defining the role of MDSCs in pancreatic cancer and/or therapy efficacy.

Mouse Models	Cells Lines	Therapeutic Agent(s)	Findings	Target(s)	Ref.
CD11b-DTR, iKras*, iKras*P53*, iKras*; CD11b-DTR, iKras*; p53*; CD11b-DTR mice	Primary human (1319, UM2, UM5, UM18 and UM19) and primary mouse (iKras*1, iKRAS*2, iKras*3, 65 671, 7940B) cell lines	–	Myeloid cell subsets favor tumor immunoevasion in EGFR-MAPK-dependent regulation of tumoral PD-L1 expression and inhibition of CD8^+^ T cell antitumor immunity	Kras^G12D^, PD-L1	[19]
Kras^LSL-G12D/+^; Cre (KC) and Kras^LSL-G12D/+^; Trp53^LSL-R172H/+^; Cre (KPC) *mice*	–	–	G-MDSCs inhibit T cell proliferation and induce T cell death and its depletion unmask PDAC to adaptive immune response.	–	[24]
EL-TGF-α/p53^−/−^ double transgenic, EL-TGF-α/p53^+/−^ heterozygous, C57BL/6 and BALB/c mice	Murine mPAC cell line	–	Increased frequency of MDSCs was detected at early stages of tumor development with further increased during tumor progression.	–	[39]
KCR, KC, RAGE-null, and C57BL/6- wild type mice	–	–	RAGE ablation resulted in the accumulation of MDSCs.	RAGE	[40]
GCSFR^−/−^, NU/J and C57BL/6-WT mice	KCM, KCKO, Panc-1, BxPC3 and Pan02 cell lines	–	STAT3 signaling in M-MDSCs promotes CSCs stemness in pancreatic cancer.	STAT3	[41]
C57BL/6 mice	Murine Panc02 cell line	Sildenafil	Sildenafil treatment reduced MDSCs frequency and VEGF levels and increased the survival of tumor-bearing female mice.	VEGF	[42]
NOD/SCID mice	PANC-1, CFPAC-1 and EL4 cell lines	Neutralizing antibodies against PAUF and TLR4, and inhibitor of the MAPK pathway	PAUF regulates the functional activation of MDSCs via TLR4 and the MAPK-dependent pathways.	PAUF	[43]
TLR4^−/−^ C57BL/6-WT and Nude mice	EL4, MSC-1 and MSC-2 cell lines	Cyclophosphamide, doxorubicin, oxaliplatin, paclitaxel, gemcitabine, 5FU, and raltitrexed	5FU-mediated depletion of MDSCs promoted CD8^+^T-cell-dependent anti-tumor responses.	–	[44]
Severe combined immunodeficiency disease (SCID) and C57BL/6-WT mice	Human Panc-1 and Murine Panc-02 cell lines	Gemcitabine, metronomic chemotherapy (MC) with gemcitabine and anti-Bv8 antibody	MC with gemcitabine or anti-Bv8 antibody enhanced gemcitabine efficacy.	–	[45]
LSL-Kras^G12D^; Pdx-1/Cre (KC) and pdx-1/Cre (Cre) mice	Murine PDA cell line	Monoclonal antibody targeting ENO1	Anti-ENO1 impaired MDSCs invasion and elicited sustained effector T-cell function.	ENO1	[46]
KPC Cxcr2^−/−^ and KPC mice	–	CXCR2 inhibitor, gemcitabine, and anti-PD1 immunotherapy	CXCR2 inhibition suppressed metastasis and enhanced therapeutic responses of chemotherapy and immunotherapy to prolong mice survival.	CXCR2	[47]
NOD/SCID mice	Human Hs766t, and MIA PaCa-2 cell lines	Serp-1, neuroserpin, and M-T7	Serp-1 and neuroserpin treatment reduced pancreatic tumor growth via decreasing splenic and tumoral MDSCs as well as tumor infiltration of macrophage.	uPA	[48]

**Table 3:** Summary of clinical studies defining the role of MDSCs in pancreatic cancer and/or therapy efficacy.

Therapeutic Agent(s)	Findings	Ref.
–	MDSCs play an essential role in pancreatic cancer but were not correlated with tumor stage.	[15]
–	MDSCs play importance role in pancreatic cancer progression	[66]
–	M-MDSCs can be characterized as circulating STAT3/arginase-1-expressing CD14+ cells in pancreatic cancer patients.	[67]
Chemotherapy + Cytokine-induced killer cell (CIK) immunotherapy	MDSCs-targeting chemotherapy improved the survival response of CIK immunotherapy.	[14]
Chemotherapy	Analysis of MDSCs in peripheral blood may represent a predictive biomarker for chemotherapy failure in pancreatic cancer patients.	[30]
Zoledronic Acid (ZA)	No differences were observed in the prevalence of G-MDSCs in the blood and bone marrow of PDAC patients treated (pre- and post) with ZA.	[68]
Gemcitabine + Capecitabine alone versus GV1001 vaccine with gemcitabine + capecitabine along with GM-CSF as adjuvant	Gemcitabine and capecitabine combination did not result in a consistent reduction in MDSCs levels. High levels of MDSCs pre-vaccination do not prevent the development of an immune response to tumor antigens.	[69]
CDDO-Me alone and CDDO-Me combination with gemcitabine	CDDO-Me abrogated the immune suppressive effects of MDSCs and improved immune response.	[70]
DS-82373a, an agonistic TRAIL-R2 antibody	DS-82373a selectively reduced MDSCs subsets in peripheral blood and tumor tissues of cancer patients, including pancreatic cancer.	[71]
